# Therapy outcomes of IL-17 and JAK inhibitors in rosacea: A systematic review

**DOI:** 10.7555/JBR.38.20240107

**Published:** 2024-08-21

**Authors:** Xinyi Dai, Chenxingyue Zhang, Zhiqiang Yin

**Affiliations:** Department of Dermatology, the First Affiliated Hospital of Nanjing Medical University, Nanjing, Jiangsu 210029, China

Dear Editor,

Rosacea is characterized by persistent or transient erythema, papules, pustules, telangiectasia, and/or phymatous lesions^[1]^. Although multiple treatments are available for rosacea, the advent of biological agents and small-molecule agents has significantly advanced our ability to target the disease more effectively^[2]^. In the current review, we summarize the outcomes of targeted therapies in rosacea, mainly focusing on interleukin (IL)-17 inhibitors and Janus kinase (JAK) inhibitors.

We performed a PubMed search on March 9, 2024, and identified 168 studies (***Supplementary Table 1***, available online), of which 11 met the inclusion criteria, representing 72 patients receiving different targeted therapies. Of these patients, 43 received targeted therapy with IL-17 inhibitors (*n* = 17, 39.5%) or JAK inhibitors (*n* = 26, 60.5%), among whom eight (18.6%) received concomitant medications (***Table 1*** and ***Supplementary Table 2*** [available online]). The remaining 29 patients received a number of other targeted medications. Because of the small sample size of other medications, we mainly analyzed therapy outcomes for IL-17 inhibitors and JAK inhibitors in patients with rosacea.

**Table 1:** Summary of therapy outcomes of IL-17 and JAK inhibitors in rosacea patients

Characteristics	IL-17 inhibitor therapy (*n*=17)	JAK inhibitor therapy (*n*=26)
Agent [*n* (%)]	Secukinumab, 17 (100.0)	Tofacitinib, 22 (84.6); Abrocitinib, 4 (15.4)
Therapy outcomes [*n* (%)]	PR, 17 (100.0)	CR, 3 (11.5); PR, 17 (65.4); no resolution, 6 (23.1)
Mean response time (months)	Not reported	CR, 0.4; PR, 1.0
Recurrence [*n* (%)]	Not reported	Yes, 7 (35.0); no, 13 (65.0)
Mean follow-up period (months)	4.0	7.0
Adverse events [*n* (%)]	Hearing impaired, 1 (5.9); diarrhea, 3 (17.6); sinus disorder, 1 (5.9); vomiting, 1 (5.9); fatigue, 3 (17.6); injection site reaction, 1 (5.9); flu-like symptoms, 1 (5.9); otitis externa, 1 (5.9); sinusitis, 1 (5.9); skin or nail infection, 4 (23.5); urinary tract infection, 1 (5.9); gastrointestinal infection, 1 (5.9); arthralgia, 1 (5.9); none, 6 (35.3); upper respiratory infection, 1 (5.9); sinus pain, 1 (5.9); sore throat, 2 (11.8); cough, 1 (5.9); dysuria, 1 (5.9); pruritus, 3 (17.6); allergic rhinitis, 1 (5.9); rash (eczema), 2 (11.8)	Not reported, 19 (73.1); none, 5 (19.2); elevated liver enzymes, 1 (3.8); elevated serum bilirubin, 1 (3.8)
Abbreviations: IL-17, interleukin-17; JAK, Janus kinase; CR, complete resolution; PR, partial resolution.		

IL-17 inhibitors showed the highest efficacy, achieving partial resolution in all 17 cases (100.0%). JAK inhibitors followed with complete resolution in three cases (11.5%) within 0.4 months, partial resolution in 17 cases (65.4%) within 1.0 months, and no resolution in six cases (23.1%) (***Table 1*** and ***Supplementary Table 3*** [available online]). There was no heterogeneity between patients with and without concomitant medications.

The pathology of rosacea is linked to immune dysfunction dominated by the Th1/Th17-polarized immune cells^[1–2]^. These T cells express elevated levels of interferon-gamma (IFN-γ), tumor necrosis factor-alpha (TNF-α), and IL-17A, which are associated with inflammation, angiogenesis, and the induction of matrix metalloproteinase-9 (MMP-9) and cathelicidin antimicrobial peptide LL37^[1–2]^. Therefore, the inhibition of IL-17 and TNF-α has shown some favorable outcomes. The importance of JAK-signal transducer and activator of transcription (STAT) signaling in the pathogenesis of rosacea is related to its effects on the skin barrier and immune cell activation^[3]^, and the inhibitors of these pathways may play a role in a valid therapeutic approach for rosacea, given the upregulation of STAT transcription factors. Additionally, our search results included some individual and small-sample targeted medications, such as phosphodiesterase inhibitors that may reduce the production of TNF-α, IL-12, IL-23, and the response of natural killer cells and keratinocytes^[4]^, anti-vascular endothelial growth factor agents that have an inhibitory effect on vascular maturation^[2,5]^, and the calcitonin gene-related peptide (CGRP) inhibitors, which may improve treatment outcomes in rosacea patients with migraine according to this analysis (***Supplementary Table 4***, available online).

Limitations include the small sample size, the lack of a unified evaluation system for rosacea resolution, and the difficulty in isolating the effects of targeted therapies because of concomitant treatments. Although additional larger studies are needed, the occurrence of adverse events also merits attention. Targeted therapies, especially IL-17 inhibitors and JAK inhibitors, may become effective adjunctive treatments for rosacea.

Yours sincerely,Xinyi Dai^△^, Chenxingyue Zhang^△^, Zhiqiang Yin^✉^ Department of Dermatology, the First Affiliated Hospital of Nanjing Medical University,Nanjing, Jiangsu 210029,China.^△^These authors contributed equally to this work.^✉^Corresponding author: Zhiqiang Yin. E-mail:yinzhiqiang@njmu.edu.cn.

## SUPPLEMENTARY DATA

Supplementary data to this article can be found online.
