# Supplementary material for: Therapy outcomes of IL-17 and JAK inhibitors in rosacea: A systematic review
Source: J Biomed Res. 2024 Aug 21;39(3):317–8. doi: 10.7555/JBR.38.20240107 (PMC12239976; doi:10.7555/JBR.38.20240107)
Supplement: Supplementary file 1 — Supplementary data to this article can be found online. [file jbr-39-3-317-Supplementary.pdf]

Available online at www.jbr-pub.org.cn
Open Access at PubMed Central
Journal of Biomedical Research, 2025 39(3): 1–3
Supplementary Data
JBR

Therapy outcomes of IL-17 and JAK inhibitors in rosacea: A systematic review

Xinyi Dai△, Chenxingyue Zhang△, Zhiqiang Yin✉

Department of Dermatology, the First Affiliated Hospital of Nanjing Medical University, Nanjing, Jiangsu 210029, China.

Supplementary Table 1 **Search strategy and database(s): PubMed**

Order of search	Keyword of searches	Number of results
1	"Rosacea"[MeSH]	3 665
2	"Rosacea"[MeSH] OR Acne Rosacea OR Phymatous Rosacea OR Rosacea, Phymatous OR Rosacea, Ocular OR Papulopustular Rosacea OR Rosacea, Papulopustular OR Erythematotelangiectatic Rosacea OR Rosacea, Erythematotelangiectatic OR Granulomatous Rosacea OR Rosacea, Granulomatous	5 144
3	"Tumor Necrosis Factor-alpha"[MeSH]	141 720
4	"Interleukin-17"[MeSH]	15 247
5	"Interleukin-23"[MeSH]	4 918
6	IL-12/23	603
7	"Janus Kinase Inhibitors"[MeSH]	1 800
8	"Phosphodiesterase 4 Inhibitors"[MeSH]	1 455
9	"Tyrosine Kinase Inhibitors"[MeSH]	536
10	VEGF	99 338
11	Adalimumab	11 502
12	Infliximab	18 210
13	Etanercept	9 893
14	Certolizumab pegol	1 268
15	Tildrakizumab	301
16	Risankizumab	485
17	Guselkumab	695
18	Ustekinumab	3 434
19	Tofacitinib	3 231
20	Abrocitinib	224
21	Apremilast	1 173
22	Imatinib	18 087
23	Sunitinib	7 665

△These authors contributed equally to this work.
✉Corresponding author: Zhiqiang Yin, Department of Dermatology, the First Affiliated Hospital of Nanjing Medical University, Nanjing, Jiangsu 210029, China. E-mail: yinzhiqiang@njmu.edu.cn.

Received: 11 April 2024; Revised: 04 August 2024; Accepted: 08 August 2024; Published online: 21 August 2024

CLC number: R758.734, Document code: B

The authors reported no conflict of interests.

This is an open access article under the Creative Commons Attribution (CC BY 4.0) license, which permits others to distribute, remix, adapt and build upon this work, for commercial use, provided the original work is properly cited.

© 2025 by Journal of Biomedical Research.

https://doi.org/10.7555/JBR.38.20240107

Table 1 **Search strategy and database(s): PubMed** (Continued)

Order of search	Keyword of searches	Number of results
24	Bevacizumab	23 752
25	Ranibizumab	6 590
26	"Immunoglobulins"[MeSH]	997 459
27	("Rosacea"[MeSH] OR Acne Rosacea OR Phymatous Rosacea OR Rosacea, Phymatous OR Rosacea, Ocular OR Papulopustular Rosacea OR Rosacea, Papulopustular OR Erythematotelangiectatic Rosacea OR Rosacea, Erythematotelangiectatic OR Granulomatous Rosacea OR Rosacea, Granulomatous) AND ("Tumor Necrosis Factor-alpha"[MeSH] OR Interleukin-17 OR Interleukin-23 OR IL-12/23 OR "Janus Kinase Inhibitors"[MeSH] OR "Phosphodiesterase 4 Inhibitors"[MeSH] OR "Tyrosine Kinase Inhibitors"[MeSH] OR VEGF OR Adalimumab OR Infliximab OR Etanercept OR Certolizumab pegol OR Tildrakizumab OR Risankizumab OR Guselkumab OR Ustekinumab OR Tofacitinib OR Abrocitinib OR Apremilast OR Imatinib OR Sunitinib OR Bevacizumab OR Ranibizumab OR "Immunoglobulins"[MeSH])	168

Supplementary Table 2 **Summary of therapy outcomes of IL-17 and JAK inhibitors categorized by concomitant medications in rosacea patients**

Variables	No concomitant medications			Concomitant medications	
	Janus kinase inhibitors		IL-17 inhibitors	Janus kinase inhibitors	
	Tofacitinib (*n*=17)	Abrocitinib (*n*=1)	Secukinumab (*n*=17)	Tofacitinib (*n*=5)	Abrocitinib (*n*=3)
CR [*n* (%)]	1 (5.9)	1 (100.0)	0	1 (20)	0
CR time (months)	0.5	0.25	NA	0.5	0
PR [*n* (%)]	10 (58.8)	0	17（100.0）	4 (80.0)	3 (100.0)
PR time (months)	1.08	0	4	1	0.5
Adverse event (*n*)	Yes, *n*=1<br>No, *n*=0<br>NR, *n*=16	Yes, *n*=0<br>No, *n*=1<br>NR, *n*=0	Yes, *n*=11<br>No, *n*=6<br>NR, *n*=0	Yes, *n*=1<br>No, *n*=1<br>NR, *n*=3	Yes, *n*=0<br>No, *n*=3<br>NR, *n*=0
Recurrence (*n*)	Yes, *n*=7<br>No, *n*=10<br>NR, *n*=0	Yes, *n*=0<br>No, *n*=1<br>NR, *n*=0	Yes, *n*=0<br>No, *n*=0<br>NR, *n*=17	Yes, *n*=0<br>No, *n*=5<br>NR, *n*=0	Yes, *n*=0<br>No, *n*=3<br>NR, *n*=0
No resolution [*n* (%)]	6 (35.3)	0	0	0	0

CR, complete resolution; PR, partial resolution; NR, not reported; NA, not applicable.

Supplementary Table 3 **Summary of targeted therapy outcomes in rosacea patients**

Variables	JAK inhibitor (tofacitinib, *n*=22)	JAK inhibitor (abrocitinib, *n*=4)	IL-17 inhibitor (secukinumab, *n*=17)	MAb to CGRP (galcanezumab, erenumab, remanezumab, *n*=13)	Phosphodiesterase inhibitor (apremilast, *n*=10)	Anti-VEGF agent (bevacizumab, *n*=4)	TNF-α inhibitor (adalimumab, *n*=2)
CR [*n* (%)]	2 (9.1)	1 (25.0)	0	0	0	0	0
CR time (months)	0.5	0.25	NA	NA	NA	NA	NA
PR [*n* (%)]	14 (63.6)	3 (75.0)	17 (100.0)	6 (46.2)	6 (60.0)	4 (100.0)	1 (50.0)
PR time (months)	0.67	0.5	4	NR	0.83	3.16	3
Adverse event (*n*)	Yes, *n*=2<br>No, *n*=1<br>NR, *n*=19	Yes, *n*=0<br>No, *n*=4<br>NR, *n*=0	Yes, *n*=11<br>No, *n*=6<br>NR, *n*=0	Yes, *n*=3<br>No, *n*=10<br>NR, *n*=0	Yes, *n*=4<br>No, *n*=6<br>NR, *n*=0	Yes, *n*=0<br>No, *n*=1<br>NR, *n*=3	Yes, *n*=0<br>No, *n*=2<br>NR, *n*=0
No resolution [*n* (%)]	6 (27.3)	0	0	7 (53.8)	4 (40.0)	0	1 (50.0)

Abbreviations: JAK, Janus kinase; IL-17, interleukin-17; MAb, monoclonal antibody; CGRP, calcitonin gene-related peptide; VEGF, vascular endothelial growth factor; TNF-α, tumor necrosis factor-alpha; CR, complete resolution; PR, partial resolution; NA, not applicable.

Supplementary Table 4 (available online) shows the study characteristics, patient characteristics, rosacea information, treatment characteristics, and outcomes of all studies included.

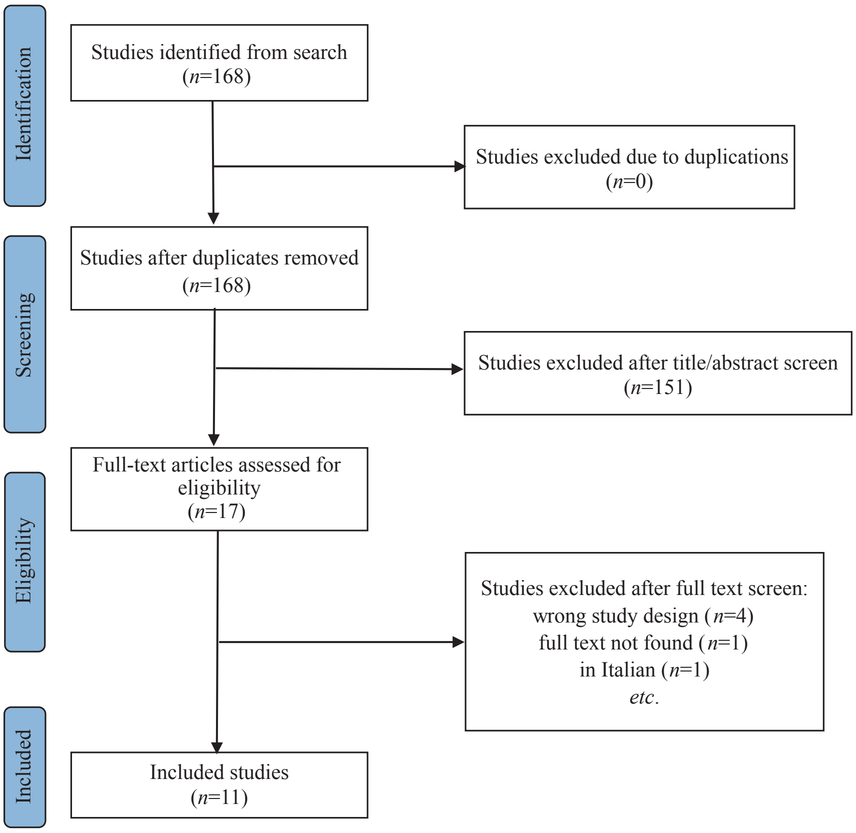

Supplementary Fig. 1 **Selection for the study inclusion.** Studies were eligible if they: (1) included patients with rosacea treated with targeted therapies, including biologics and small-molecule agents; (2) were observational or experimental studies involving human subjects; and (3) had data in the English language.
